# Proteomic analysis of scallop hepatopancreatic extract provides insights into marine polysaccharide digestion

**DOI:** 10.1038/srep34866

**Published:** 2016-12-16

**Authors:** Qianqian Lyu, Wenqian Jiao, Keke Zhang, Zhenmin Bao, Shi Wang, Weizhi Liu

**Affiliations:** 1 Ministry of Education Key Laboratory of Marine Genetics and Breeding, College of Marine Life Sciences, Ocean University of China, Qingdao, China; 2 Laboratory for Marine Biology and Biotechnology, Qingdao National Laboratory for Marine Science and Technology, Qingdao, China

## Abstract

Marine polysaccharides are used in a variety of applications, and the enzymes that degrade these polysaccharides are of increasing interest. The main food source of herbivorous marine mollusks is seaweed, and several polysaccharide-degrading enzymes have been extracted from mollusk digestive glands (hepatopancreases). Here, we used a comprehensive proteomic approach to examine the hepatopancreatic proteins of the Zhikong scallop (*Chlamys farreri*). We identified 435 proteins, the majority of which were lysosomal enzymes and carbohydrate and protein metabolism enzymes. However, several new enzymes related to polysaccharide metabolism were also identified. Phylogenetic and structural analyses of these enzymes suggest that these polysaccharide-degrading enzymes may have a variety of potential substrate specificities. Taken together, our study characterizes several novel polysaccharide-degrading enzymes in the scallop hepatopancreas and provides an enhanced view of these enzymes and a greater understanding of marine polysaccharide digestion.

Seaweeds are a major resource in the marine ecosystem, and they contain various polysaccharides, including alginate, mannan, cellulose and laminarin. Marine algal polysaccharides are distinct from terrestrial forms in both their diversity and their modifications^1^. For example, sulfated polysaccharides are found in brown marine seaweeds, which may be related to the high salinity of their environment^2^. Notably, seaweed polysaccharides and their corresponding degradation products exhibit various biomedical properties, including antitumor, antihypertensive, and antioxidant activities^3^,^4^,^5^,^6^. The biomedical activities of seaweed polysaccharides are a function of their monosaccharide composition, molecular weight, and sulfate content. Lower-molecular-weight oligosaccharides have discrete advantages over longer polysaccharides in pharmaceutical uses^6^,^7^. Consequently, the identification of novel enzymes capable of digesting seaweed polysaccharides is important.

For some herbivorous marine mollusks, seaweeds provide not only a habitable environment but also their daily diets. Therefore, these marine mollusks likely possess a breadth of polysaccharide-degrading enzymes with unique substrate specificities and mechanisms of action^8^,^9^. Indeed, a number of polysaccharide-degrading enzymes, including alginate lyase, mannanase, cellulose and laminarinase, have been derived from the digestive tracts of marine mollusks^10^,^11^,^12^,^13^. These enzymes exhibit high cleavage efficiency and unique substrate specificity, suggesting that marine mollusks could be a source of novel polysaccharide-degrading enzymes.

In the current study, we focused on the Zhikong scallop (*Chlamys farreri*), which is widely distributed in the oceans around north of China. Scallops are filter feeders and eat plankton, including macroalgae fragments, microalgae, bacteria and copepods. In a previous study, scallop (*Chlamys albidus* and *Mizuhopecten yessoensis*) endo-β-1,3-glucanases were extracted and characterized^14^,^15^, and a novel α-L-fucosidase was isolated from the scallop *Pecten maximus*^16^. However, although several polysaccharide-degrading enzymes have been identified from the scallop hepatopancreas, the diversity of these scallop enzymes remains unclear. To explore a more comprehensive method for identifying scallop hepatopancreas polysaccharidases, we carried out a proteomic approach to identify these enzymes. A variety of proteins were identified, including several distinct polysaccharide-degrading enzymes and enzymes associated with sulfate catabolism. Our study provides new information relevant to the enzymatic adaptation of marine algal diets and a description of novel marine polysaccharide-degrading enzymes.

## Results and Discussion

### Mass spectroscopy analysis

**Figure 1:**
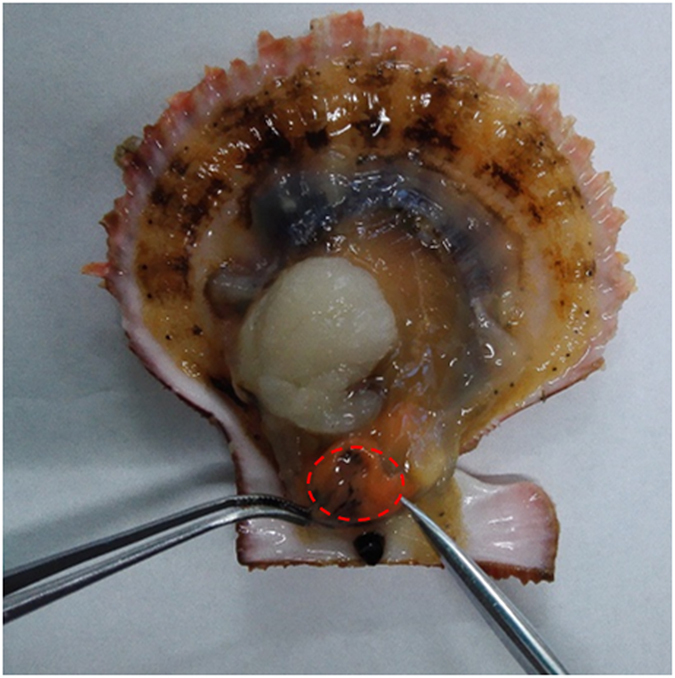
Photograph of scallop hepatopancreas. The scallop hepatopancreas is marked by a dashed circle.

**Figure 2:**
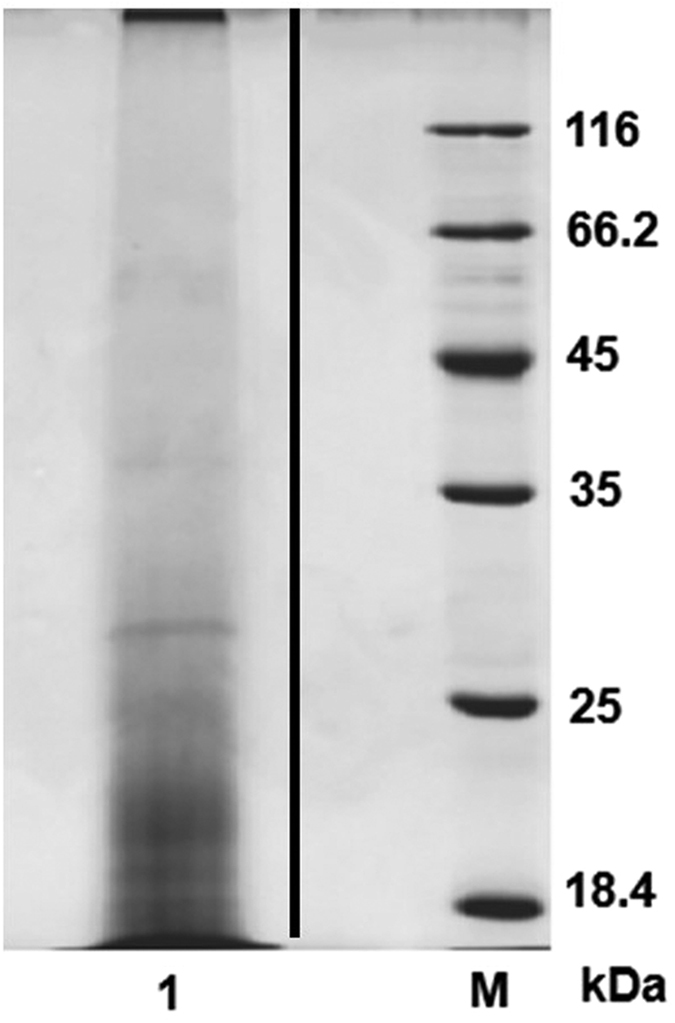
SDS-PAGE analysis scallop hepatopancreas extract. Lane 1, Scallop hepatopancreas extract; lane M, protein marker.

Proteins extracted from the scallop digestive gland were subjected to SDS-PAGE (Figs 1 and 2), and the protein mixtures were analyzed using a shot-gun proteomics approach as described in the Materials and Methods section. LC-MS/MS analysis identified 477 unique proteins, and 435 unique proteins could be annotated using BlastX against the Swiss-prot database (Supplementary Table S1). These analyses represent a comprehensive protein profile for the scallop hepatopancreas.

Gene ontology (GO) term annotation and enrichment analysis of the scallop hepatopancreatic proteins demonstrated that the most enriched GO categories were metabolic and biological processes, including 109 proteins with hydrolytic activity (GO: 0016787) (P value: 5.72E-22) and 164 proteins associated with organic substance metabolic processes (GO: 0071704) (P value: 9.15E-15) (Table 1). Both of these GO categories were dominated by glycoside hydrolases, sulfatases, proteinases and components of the proteasome, previously reported as lysosomal enzymes^17^ (Table 1).

Kyoto Encyclopedia of Genes and Genomes (KEGG) analysis indicated these hepatopancreatic proteins were significantly enriched in pathways involved in glycan degradation and the biosynthesis of amino acids as well as lysosomal and proteasomal pathways. Many of the proteins associated with these pathways contribute to carbohydrate and protein metabolism (Table 2). The proteins identified correlate well with the biological function of the hepatopancreas, the main digestive organ in scallops.

### Sequence analysis of the enzymes relevant to marine polysaccharide metabolism

The composition of marine polysaccharides is complex and diverse^18^. For example, agar is mainly composed of repetitive units of β-D-galactose and 3,6-anhydro-α-L-galactose^19^, alginate derived from brown algae is mainly composed of heteropolysaccharides of α-L-guluronate and β-D-mannuronate^20^, and the carrageenans derived from red algae consist of galactopyranose units^21^. Marine organisms require a variety of enzymes with different substrate specificities to digest and obtain these nutrients. In the scallop hepatopancreas, we identified mannosidase, α-glucosidase, β-galactosidase, endoglucanase, β-glucuronidase, chitotriosidase, xylose isomerase and α-L-fucosidase (Table 3). With the exception of endoglucanase and α-L-fucosidase^14^,^15^,^16^, this is the first description of these enzymes being isolated from the scallop hepatopancreas. Notably, for some of these types of enzymes, more than one novel enzyme was found (Table 3), and further analysis suggests these enzymes are likely to help the scallop digest diverse food sources as described below.

**Figure 3:**
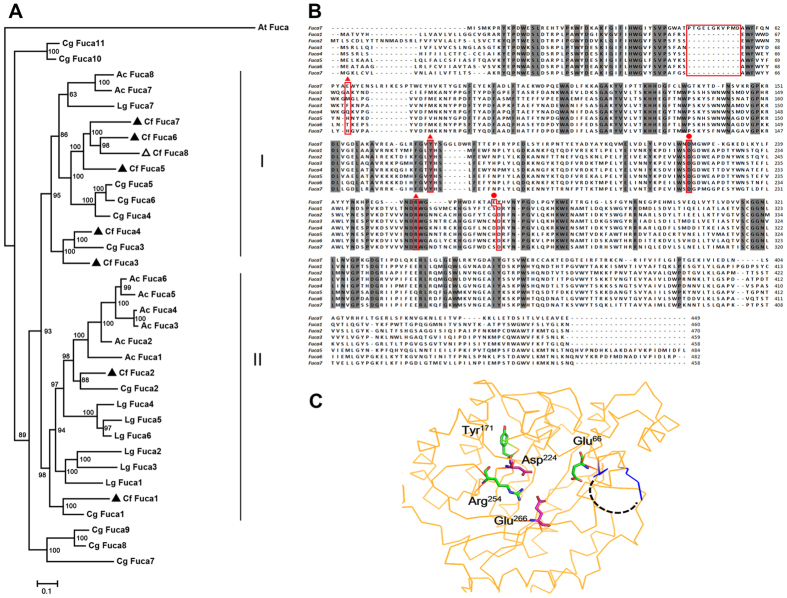
Scallop hepatopancreas α-L-fucosidase analyses. (**A**) Phylogenetic analysis of α-L-fucosidases. The *C. farreri* (Cf) sequences are highlighted by black triangles in both the genome data and proteomic analyses. Hollow triangles represent sequences present only in the genome data. Fuca: α-L-fucosidase; Cg: *Crassostrea gigas*; Lg: *Lottia gigantean*; Ac: *Aplysia californica*; At: *Arabidopsis thaliana*. The accession numbers of the protein sequences are provided in Supplementary Table S2. (**B)** Amino acid sequence alignment of the α-L-fucosidases. Identical residues are shaded in gray. *Chlamys farreri* (Fuca1–7) and *Thermotoga maritime* (FucaT, PDB: 1HL8). The two catalytic residues identified in FucaT^22^ are marked with closed circles and the three residues that form the substrate binding pocket are marked with triangles. The gap described in the text is located in the boxed region. (**C**) Structure of FucaT. The FucaT structure from *Thermotoga maritime* (PDB: 1HL8) is illustrated with yellow ribbons and the key residues shown as sticks. The loop adjacent to the substrate binding pocket is highlighted by a dashed line. The image was generated by PyMOL^32^.

Eight α-L-fucosidase genes were identified in the scallop genome, and we detected seven of these in our mass spectroscopy analysis of hepatopancreatic proteins (Table 3). These results suggest that most α-L-fucosidase genes in the genome are actually expressed and the corresponding enzymes are present in the hepatopancreas. The inability to identify the remaining α-L-fucosidase by mass spectroscopy could be due to a low expression level in the hepatopancreas. We carried out phylogenetic analyses of α-L-fucosidases from the Zhikong scallop (*C. farreri*) and other mollusk species (including the Pacific oyster, *Cassostreas gigas*, sea hare, *Aplysia californica*, and owl limpet, *Lottia gigantea*). The resulting MrBayes phylogenetic tree reveals two major molluscan clades of α-L-fucosidases (Fig. 3A). Both clades contain sequences from all of the selected mollusks. Differential duplication of α-L-fucosidases was observed for bivalves and gastropods, with the former predominantly occurring in clade I and the latter in clade II. For example, six out of eight scallop α-L-fucosidases were clustered into clade I, including Cf Fuca 3–Cf Fuca 8. Similarly, four of eleven oyster α-L-fucosidases were clustered in clade I, while only two were clustered in clade II. Similar preferential duplications were also detected for the gastropods (sea hare and owl limpet) in clade II (Fig. 3A). The high diversity and preferential gene duplication of α-L-fucosidases in mollusks may be related to their adaptations to different food sources.

Additional sequence and structural analyses of the seven scallop hepatopancreatic α-L-fucosidases identified in the proteomic screening suggest these enzymes likely have distinct substrate binding specificities, based on their sequence conservation and substrate binding cleft analysis, as shown in Fig. 3B,C. The α-L-fucosidases are able to catalyze the removal of nonreducing terminal L-fucose residues linked by α-1,2, α-1,3, α-1,4 or α-1,6 bonds to oligosaccharides and their conjugates. They play crucial roles in the metabolic processing of fucosylated glycoconjugates, which are widely distributed from bacteria to human^22^. A previous structural analysis identified the catalytic nucleophiles Asp^224^ and Glu^266^ in a *Thermotoga maritime* α-L-fucosidase^22^. Sequence alignment of seven α-L-fucosidases identified in the scallop hepatopancreatic extract (Table 3) illustrates that these α-L-fucosidases share high sequence identity (more than 70% sequence identity); the residue Asp^224^ is highly conserved, whereas the Glu^266^ residue is not as conserved (Fig. 3B). However, an Asp residue next to that position is possibly substituted for the key Glu^266^ residue found in the *Thermotoga maritime* α-L-fucosidase, which is assumed to play a role in the completion of the reaction (Fig. 3B). In the published structure of the α-L-fucosidase from *Thermotoga maritime* (PDB: 1HL8), three residues (Glu^66^, Tyr^171^, Arg^254^) contribute to forming the substrate binding pocket (Fig. 3C). Tyr^171^ and Arg^254^ are highly conserved in all the α-L-fucosidases from the scallop hepatopancreas, whereas the Glu^66^ and neighboring residues are not conserved (highlighted in Fig. 3B,C). In addition, compared to the previously determined structure (PDB: 1HL8), the region from Pro^46^ to Asp^56^ was absent in the seven α-L-fucosidases identified in the scallop hepatopancreas. As shown in the determined α-L-fucosidase structure (Fig. 3C), this loop is adjacent to the substrate binding pocket, suggesting this region may be associated with substrate binding. Taken together, based on these observed differences in the substrate binding cleft, it is possible to speculate that the α-L-fucosidases from the scallop hepatopancreas have a distinct substrate binding specificity (Fig. 3B,C).

The presence of sulfated polysaccharides is a significant feature of seaweed as compared to terrestrial plants. Sulfatases catalyze the hydrolysis of sulfuric acid esters from a wide variety of substrates, including glycosaminoglycans, glycolipids and steroids^23^. Therefore, sulfatases play important roles in polysaccharide metabolism.

**Figure 4:**
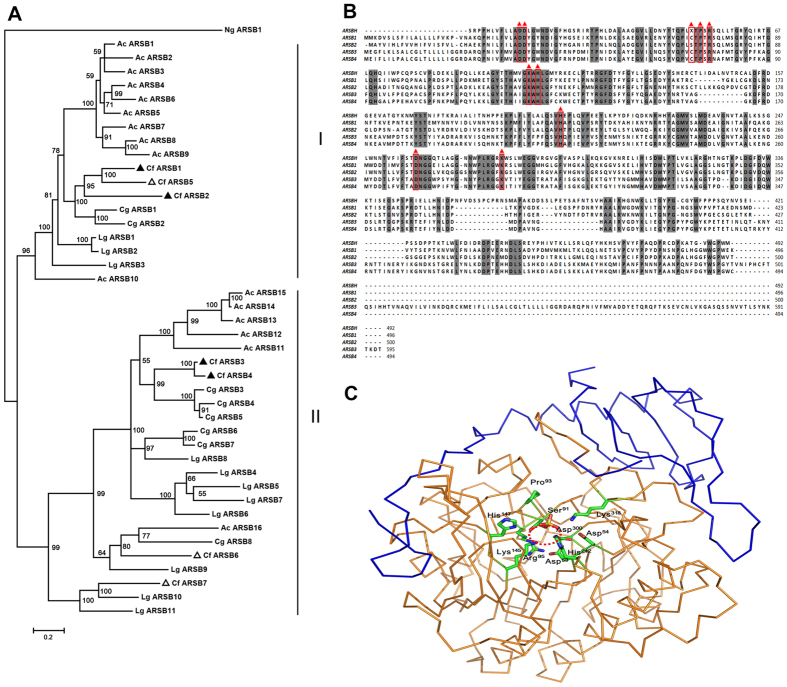
Analysis of scallop hepatopancreas arylsulfatases. (**A**) Phylogenetic analysis of arylsulfatases. The *C. farreri* (Cf) sequences are highlighted by black triangles in both the genome data and proteomic analyses. Hollow triangles represent sequences present only in the genome data. ARSB: arylsulfatase; Cg: *Crassostrea gigas*; Lg: *Lottia gigantean*; Ac: *Aplysia californica*; Ng: *Nannochloropsis gaditana* CCMP526. The accession numbers of the protein sequences are provided in Supplementary Table S2. (**B**) Amino acid sequence alignment of arylsulfatases. *C. farreri* (ARSB1–4) and human (ARSBH, PDB: 1FSU). Identical residues are shaded in gray. The ten primary active-site residues described in ARSBH are marked with triangles. (**C**) Structural analysis of ARSBH. The human structure of ARSBH (PDB: 1FSU) illustrated with orange ribbons and with the C-terminal region in blue. The primary active-site residues are shown as sticks, and the catalytic center is highlighted by a dashed circle. The picture was generated by PyMOL^32^.

Seven arylsulfatase B genes were identified in the scallop genome, four of which were also detected in protein mass spectroscopy of the scallop hepatopancreas; undetected arylsulfatases may be possibly related to a low expression level as mentioned above. Phylogenetic analysis of arylsulfatase B genes was carried out for Zhikong scallop (*C. farreri*) and other mollusk species. As shown in Fig. 4A, all molluscan arylsulfatase B proteins were clustered into two large clades. Gene duplications were observed for bivalves and gastropods in both clades. For example, duplication of Cf ARSB1, Cf ARSB2 and Cf ARSB5 occurred in clade I and duplication of Cf ARSB3, Cf ARSB4, Cf ARSB6 and Cf ARSB7 occurred in clade II. The sea hare *A. californica* had the most gene copies among the examined mollusks, with 10 copies in clade I and 6 copies in clade II. We hypothesize that the high diversity of arylsulfatase B genes observed here may be a mechanism for mollusks to adapt to diverse algae resources.

Sequence alignments and structure-based analyses were carried out to further explore the potential substrate binding specificities of the arylsulfatases. As shown in Fig. 4B, ten primary active-site residues (Asp^53^; Asp^54^; Cys^91^ (or Ser); Pro^93^; Arg^95^; Lys^145^; His^147^; His^242^; Asp^300^; and Lys^318^) (PDB: 1FSU) are highly conserved in the sulfatase family^24^. Structural analyses of arylsulfatases indicated that all of the members have positively charged active-site pockets suitable for recognizing a sulfated substrate (Fig. 4C), although the overall sizes, shapes, and electrostatics of the pockets vary extensively. In addition, the C-terminal regions of arylsulfatases play an important role in defining the active-site pocket^23^,^24^. Sequence alignment of arylsulfatases indicates that the C-terminal regions of the four arylsulfatases from *C. farreri* are not conserved and diverge significantly (Fig. 4B), which suggests they may have unique substrate specificities.

Taken together, our phylogenetic and structural analyses suggest that gene duplications of α-L-fucosidase and arylsulfatase occurred in the scallop genome along with sequence and structural variations that may possibly confer different substrate specificities to the enzymes, consistent with the diversity of phytoplankton consumed by the scallop.

## Conclusion

Proteomic approaches were applied to analyze the protein composition of the scallop hepatopancreas. A variety of enzymes associated with polysaccharide metabolism were identified, suggesting a complex enzyme system is required for the scallop to deal with diverse seaweed food sources. In support of this hypothesis, phylogenetic and structural analyses revealed gene duplications and potentially diversified protein binding specificities. Overall, our study characterizes several novel polysaccharide-degrading enzymes in the scallop hepatopancreas, providing an enhanced view of these enzymes and our understanding of marine polysaccharide digestion.

## Methods

### Soluble fractions extract from scallop hepatopancreas

Scallops were purchased from a local seafood market in Qingdao. The hepatopancreas was dissected by hand from the scallop viscera (Fig. 1), immediately transferred to the cold 1 × PBS buffer, and homogenized manually at 4 °C. The homogenate was centrifuged for 30 min at 8,000 × g, and the supernatant was subjected to ammonium sulfate fractionation. The precipitates formed under 80% ammonium sulfate were collected by centrifugation at 8,000 × g for 30 min. The precipitates were then dissolved in 1 × PBS buffer and dialyzed against 1 × PBS buffer at 4 °C overnight, and the resulting solution was lyophilized to protein powder for subsequent analysis.

### Mass spectroscopy

#### Sample preparation and SDS-PAGE

The extracted and lyophilized protein powder was dissolved in 0.4 ml SDT lysis buffer (4% SDS, 150 mM Tris-HCl, 100 mM DTT, pH 7.6)^25^, heated at 100 °C for 3 min, and then sonicated at 50 W for 5 min (2 s sonicate, 8 s rest) on ice. The sample was reheated to 100 °C for 3 min and then centrifuged at 14,000 × g for 40 min. The supernatant solution was collected and filtered, the protein concentration was determined using the Bradford method, and an aliquot of the treated sample was analyzed by SDS-PAGE (Fig. 2).

#### Filter-aided proteome enzyme solution

Protein digestion was conducted using the FASP procedure^25^. For the treated sample, 200 μg of protein was loaded onto an ultrafiltration filter (30 kDa cutoff, Sartorius, Germany) containing 200 μl of UA buffer (8 M urea, 150 mM Tris-HCl, pH 8.0) followed by centrifugation at 14,000 × g for 30 min and an additional washing step with 200 μl of UA buffer. One hundred microliters of 50 mM iodoacetamide in UA buffer was subsequently added to the filter to block the reduced cysteine residues, the sample was incubated for 30 min at room temperature in the dark, and then the sample was centrifuged at 14,000 × g for 30 min. The filters were washed twice with 100 μl of UA buffer and centrifuged at 14,000 × g for 20 min after each washing step. Next, 100 μl of 25 mM ammonium bicarbonate was added to the filter, followed by centrifugation at 14,000 × g for 20 min, which was repeated twice. The protein suspensions were then digested with 40 μl of trypsin (Promega, Madison, WI, USA) buffer (4 μg trypsin in 100 μl ammonium bicarbonate) at 37 °C for 16–18 h. Finally, the filter unit was transferred to a new tube and centrifuged at 14,000 × g for 30 min. The resulting peptides were collected as a filtrate, and the peptide concentration was analyzed at OD_280_.

#### Capillary high-performance liquid chromatography

The samples were analyzed using an Easy-nLC nanoflow HPLC system connected to an Orbitrap Elite mass spectrometer (Thermo Fisher Scientific, San Jose, CA, USA). A total of 1 μg of each sample was loaded onto a Thermo Scientific EASY column (two columns) using an autosampler at a flow rate of 150 nl/min. The sequential separation of peptides on the Thermo Scientific EASY trap column (10 μm × 2 cm, 5 μm, 100 Å, C18) and analytical column (75 μm × 25 cm, 5 μm, 100 Å, C18) was accomplished using a segmented 1 hr gradient from Solvent A (0.1% formic acid in water) to 50% Solvent B (0.1% formic acid in 100% ACN) for 50 min, followed by 50–100% Solvent B for 4 min and then 100% Solvent B for 6 min. The column was re-equilibrated to its initial highly aqueous solvent composition before each analysis.

#### Electrospray ionization mass spectrometry

The mass spectrometer was operated in positive ion mode, and MS spectra were acquired over a range of 300–1800 m/z. The resolving powers of the MS scan and MS/MS scan at 200 m/z for the Orbitrap Elite were set as 70,000 and 17,500, respectively. The top ten most intense signals in the acquired MS spectra were selected for further MS/MS analysis. The isolation window was 2 m/z, and ions were fragmented through higher energy collisional dissociation with normalized collision energies of 27 eV. The maximum ion injection times were set at 10 ms for the survey scan and 60 ms for the MS/MS scans, and the automatic gain control target values were set to 1.0 × 10^−6^ for full scan modes and 5 × 10^4^ for MS/MS. The dynamic exclusion duration was 40 s.

#### ESI MS data analysis

The raw files were analyzed using the Proteome Discoverer 1.4 software (Thermo Fisher Scientific). A search for the fragmentation spectra was performed using the MASCOT search engine embedded in Proteome Discoverer against the whole-genome database (unpublished data). The following search parameters were used: monoisotopic mass, trypsin as the cleavage enzyme, two missed cleavages, carbamidomethylation of cysteine as fixed modifications, and peptide charges of 2+, 3+, and 4+, and the oxidation of methionine, Phospho, Gln->pyro-Glu (N-terminal Gln) and Acetyl (protein N terminus) were specified as variable modifications. The mass tolerance was set to 10 ppm for precursor ions and to 0.05 Da for the fragment ions. The results were filtered based on a false discovery rate (FDR) of no more than 1%.

### Bioinformatics analysis

#### Functional annotation and enrichment

The related protein sequences and their functional annotations were retrieved from our ongoing whole-genome sequencing project for *C. farreri* (unpublished). Gene ontology (GO) enrichment analyses for proteins from the scallop hepatopancreas were carried out based on the algorithm implemented in GOstat^26^, using the whole annotated *C. farreri* gene set as the background. GO terms that were enriched within a given gene set were extracted with EnrichPipeline^27^. Similar techniques were used for the Kyoto Encyclopedia of Genes and Genomes (KEGG) enrichment analysis.

#### Multiple sequence alignment and phylogenetic analysis

The related protein sequences of *C. farreri* were obtained from the scallop genome as described above. Protein sequences from other mollusks and outgroup species were retrieved from the NCBI or Ensembl databases (accession numbers provided in Supplementary Table S2). Amino acid sequences were aligned using Clustal W version 2.0^28^ and alignments were adjusted manually. Only unambiguously aligned positions (selected by the program GBlocks^29^,^30^) were considered in phylogenetic analyses. Phylogenetic trees were constructed using MrBayes v3.2^31^. The MCMC chain was allowed to run for 10^6^ generations saving one tree every 100 generations. The first 25% of the resulting samples were discarded, and the remaining trees were used to construct a majority rule consensus tree, with a posterior probability of 0.95 or greater considered significant.

## Additional Information

**How to cite this article**: Qianqian, L. *et al*. Proteomic analysis of scallop hepatopancreatic extract provides insights into marine polysaccharide digestion. *Sci. Rep.* **6**, 34866; doi: 10.1038/srep34866 (2016).

**Publisher's note:** Springer Nature remains neutral with regard to jurisdictional claims in published maps and institutional affiliations.

## Supplementary Material

Supplementary Dataset 1

Supplementary Information

## Figures and Tables

**Table 1:** GO term enrichment of scallop hepatopancreas proteins.

GO ID	GO Term	GO Class	Gene number	P value	Adjusted Pv
GO:0016787	hydrolase activity	MF	109	5.72E-22	7.24E-20
GO:0071704	organic substance metabolic process	BP	164	9.15E-15	5.80E-13
GO:0044238	primary metabolic process	BP	151	2.51E-12	1.31E-10
GO:0044710	single-organism metabolic process	BP	85	6.28E-09	1.69E-07
GO:0016491	oxidoreductase activity	MF	50	4.34E-08	9.29E-07
GO:0030529	ribonucleoprotein complex	CC	21	3.88E-07	6.62E-06
GO:0003735	structural constituent of ribosome	MF	19	6.79E-07	1.14E-05
GO:0044424	intracellular part	CC	68	6.70E-06	9.20E-05
GO:0065008	regulation of biological quality	BP	12	9.96E-06	0.000128082
GO:0005615	extracellular space	CC	5	5.91E-05	0.00060889
GO:0030117	membrane coat	CC	6	9.17E-05	0.000855833
GO:0048475	coated membrane	CC	6	9.17E-05	0.000855833
GO:0043228	non-membrane-bounded organelle	CC	28	0.000585	0.003787825
GO:0016853	isomerase activity	MF	8	0.0007829	0.004890607
GO:0061134	peptidase regulator activity	MF	7	0.0022176	0.012067567
GO:0009056	catabolic process	BP	22	0.0023802	0.012757275
GO:0043234	protein complex	CC	28	0.0028176	0.014769847
GO:0004857	enzyme inhibitor activity	MF	7	0.0038425	0.019118285
GO:0031982	vesicle	CC	4	0.0038797	0.019118285
GO:0030119	AP-type membrane coat adaptor complex	CC	3	0.0053317	0.02388487
GO:0005622	intracellular	CC	72	0.0065705	0.028323924
GO:0004601	peroxidase activity	MF	5	0.0146759	0.054239627

**Table 2:** KEGG enrichment of scallop hepatopancrea proteins.

MapID	MapTitle	Gene number	P value	Adjusted Pv
04142	Lysosome	43	2.40E-41	6.06E-39
00511	Other glycan degradation	13	1.00E-08	1.26E-06
01230	Biosynthesis of amino acids	17	4.42E-08	3.11E-06
03050	Proteasome	12	4.94E-08	3.11E-06
01200	Carbon metabolism	18	8.63E-08	4.35E-06
04612	Antigen processing and presentation	12	4.06E-07	1.71E-05
00710	Carbon fixation in photosynthetic organisms	9	6.23E-07	2.24E-05
00030	Pentose phosphate pathway	9	2.89E-06	9.10E-05
00051	Fructose and mannose metabolism	10	2.09E-05	0.000586
00010	Glycolysis/Gluconeogenesis	11	3.98E-05	0.001002
00531	Glycosaminoglycan degradation	9	6.39E-05	0.001464
03010	Ribosome	21	0.00013	0.002408
00053	Ascorbate and aldarate metabolism	11	0.000134	0.002408
05203	Viral carcinogenesis	17	0.000297	0.004993
00930	Caprolactam degradation	5	0.000341	0.005371

**Table 3:** Polysaccharide metabolism related enzymes identified in scallop hepatopancreatic extract by mass spectroscopy.

Type	Enzyme number
α-mannosidase	1
β- mannosidase	1
α-glucosidase	1
β-glucuronidase	1
α-L-fucosidase	7
α-galactosidase	1
β-galactosidase	2
Arylsulfatase	4
Endoglucanase	1
N-acetylgalactosamine-6-sulfatase	2
Chitotriosidase	1

## References

[b1] Alves A., Caridade S. G., Mano J. F., Sousa R. A. & Reis R. L. Extraction and physico-chemical characterization of a versatile biodegradable polysaccharide obtained from green algae. Carbohydr. Res. 345, 2194–2200 (2010). 20800225 10.1016/j.carres.2010.07.039

[b2] Aquino R. S., Grativol C. & Mourão P. A. Rising from the sea: correlations between sulfated polysaccharides and salinity in plants. PloS one 6, 508–508 (2011). 10.1371/journal.pone.0018862 PMC3084243 21552557

[b3] Jiao L., Jiang P., Zhang L. & Wu M. Antitumor and immunomodulating activity of polysaccharides from Enteromorpha intestinalis. Biotechnol. Bioprocess Eng. 15, 421–428 (2010).

[b4] Qi H. & Sun Y. Antioxidant activity of high sulfate content derivative of ulvan in hyperlipidemic rats. Int. J. Biol. Macromol. 76, 326–329 (2015). 25773592 10.1016/j.ijbiomac.2015.03.006

[b5] Choi D.-S. . Antioxidant activity of sulfated polysaccharides isolated from Sargassum fulvellum. Prev. Nutr. Food Sci. 12, 65–73 (2007).

[b6] Mak W., Hamid N., Liu T., Lu J. & White W. Fucoidan from New Zealand Undaria pinnatifida: Monthly variations and determination of antioxidant activities. Carbohydr. Polym. 95, 606–614 (2013). 23618312 10.1016/j.carbpol.2013.02.047

[b7] Moroney N. C., O’Grady M. N., Lordan S., Stanton C. & Kerry J. P. Seaweed Polysaccharides (Laminarin and Fucoidan) as Functional Ingredients in Pork Meat: An Evaluation of Anti-Oxidative Potential, Thermal Stability and Bioaccessibility. Mar. Drugs 13, 2447–2464 (2015). 25903283 10.3390/md13042447 PMC4413220

[b8] Trincone A. Marine enzymes for biocatalysis: sources, biocatalytic characteristics and bioprocesses of marine enzymes. 333–360 (Woodhead, 2013).

[b9] Johnston D., Moltschaniwskyj N. & Wells J. Development of the radula and digestive system of juvenile blacklip abalone (Haliotis rubra): potential factors responsible for variable weaning success on artificial diets. Aquaculture 250, 341–355 (2005).

[b10] Rahman M. M., Inoue A., Tanaka H. & Ojima T. Isolation and characterization of two alginate lyase isozymes, AkAly28 and AkAly33, from the common sea hare Aplysia kurodai. Comp. Biochem. Physiol. B Biochem. Mol. Biol. 157, 317–325 (2010). 20708706 10.1016/j.cbpb.2010.07.006

[b11] Ootsuka S., Saga N., Suzuki K.-i., Inoue A. & Ojima T. Isolation and cloning of an endo-β-1, 4-mannanase from Pacific abalone Haliotis discus hannai. J. Biotechnol. 125, 269–280 (2006). 16621092 10.1016/j.jbiotec.2006.03.008

[b12] Suzuki K. i., Ojima T. & Nishita K. Purification and cDNA cloning of a cellulase from abalone Haliotis discus hannai. Eur. J. Biochem. 270, 771–778 (2003). 12581217 10.1046/j.1432-1033.2003.03443.x

[b13] Kumagai Y., Inoue A., Tanaka H. & Ojima T. Preparation of β‐1, 3‐glucanase from scallop mid‐gut gland drips and its use for production of novel heterooligosaccharides. Fish. Sci. 74, 1127–1136 (2008).

[b14] Kovalchuk S. N. . An endo-(1→3)-β-D-glucanase from the scallop Chlamys albidus: catalytic properties, cDNA cloning and secondary-structure characterization. Carbohydr. Res. 344, 191–197 (2009). 19026410 10.1016/j.carres.2008.10.028

[b15] Kovalchuk S. N. . Purification, cDNA cloning and homology modeling of endo-1, 3-β-D-glucanase from scallop Mizuhopecten yessoensis. Comp. Biochem. Physiol. B Biochem. Mol. Biol. 143, 473–485 (2006). 16473536 10.1016/j.cbpb.2005.12.024

[b16] Berteau O., McCort I., Goasdoué N., Tissot B. & Daniel R. Characterization of a new α-L-fucosidase isolated from the marine mollusk Pecten maximus that catalyzes the hydrolysis of α-L-fucose from algal fucoidan (Ascophyllum nodosum). Glycobiology 12, 273–282 (2002). 12042250 10.1093/glycob/12.4.273

[b17] Siva Kumar N. & Bhamidimarri M, P. Lysosomal Enzymes and their Receptors in Invertebrates: An Evolutionary Perspective. Curr. Protein Pept. Sci. 16, 49–65 (2015). 25692847 10.2174/1389203716666150213161311

[b18] Wang L., Wang X., Wu H. & Liu R. Overview on biological activities and molecular characteristics of sulfated polysaccharides from marine green algae in recent years. Mar. Drugs 12, 4984–5020 (2014). 25257786 10.3390/md12094984 PMC4178480

[b19] Duckworth M. & Yaphe W. The structure of agar: Part I. Fractionation of a complex mixture of polysaccharides. Carbohydr. Res. 16, 189–197 (1971).

[b20] Inoue A. . Characterization of an alginate lyase, Flalya, from Flavobacterium sp. strain UMI-01 and its expression in Escherichia coli. Mar. Drugs 12, 4693–4712 (2014). 25153766 10.3390/md12084693 PMC4145338

[b21] de Jesus Raposo M. F., de Morais A. M. B. & de Morais R. M. S. C. Marine polysaccharides from algae with potential biomedical applications. Mar. Drugs 13, 2967–3028 (2015). 25988519 10.3390/md13052967 PMC4446615

[b22] Sulzenbacher G. . Crystal Structure of Thermotoga maritima α-L-Fucosidase insights into the catalytic mechanism and the molecular basis for fucosidosis. J. Biol. Chem 279, 13119–13128 (2004). 14715651 10.1074/jbc.M313783200

[b23] Bond C. S. . Structure of a human lysosomal sulfatase. Structure 5, 277–289 (1997). 9032078 10.1016/s0969-2126(97)00185-8

[b24] Rivera-Colón Y., Schutsky E. K., Kita A. Z. & Garman S. C. The structure of human GALNS reveals the molecular basis for mucopolysaccharidosis IV A. J. Mol. Biol. 423, 736–751 (2012). 22940367 10.1016/j.jmb.2012.08.020 PMC3472114

[b25] Wisniewski J. R., Zougman A., Nagaraj N. & Mann M. Universal sample preparation method for proteome analysis. Nat. methods 6, 359–362 (2009). 19377485 10.1038/nmeth.1322

[b26] Beißbarth T. & Speed T. P. GOstat: find statistically overrepresented Gene Ontologies within a group of genes. Bioinformatics 20, 1464–1465 (2004). 14962934 10.1093/bioinformatics/bth088

[b27] Chen S. . De novo analysis of transcriptome dynamics in the migratory locust during the development of phase traits. PloS one 5, 5–12 (2010). 10.1371/journal.pone.0015633 PMC3012706 21209894

[b28] Thompson J. D., Higgins D. G. & Gibson T. J. CLUSTAL W: improving the sensitivity of progressive multiple sequence alignment through sequence weighting, position-specific gap penalties and weight matrix choice. Nucleic. Acids Res. 22, 4673–4680 (1994). 7984417 10.1093/nar/22.22.4673 PMC308517

[b29] Castresana J. Selection of conserved blocks from multiple alignments for their use in phylogenetic analysis. Mol. Biol. Evol. 17, 540–552 (2000). 10742046 10.1093/oxfordjournals.molbev.a026334

[b30] Talavera G. & Castresana J. Improvement of phylogenies after removing divergent and ambiguously aligned blocks from protein sequence alignments. Syst. Biol. 56, 564–577 (2007). 17654362 10.1080/10635150701472164

[b31] Ronquist F. & Huelsenbeck J. P. MrBayes 3: Bayesian phylogenetic inference under mixed models. Bioinformatics 19, 1572–1574 (2003). 12912839 10.1093/bioinformatics/btg180

[b32] Delano W. L. & Delano W. L. The PyMOL Molecular Graphics System. My Publications (2002).

