# Supplementary material for: Proteomic analysis of scallop hepatopancreatic extract provides insights into marine polysaccharide digestion
Source: Sci Rep. 2016 Dec 16;6:34866. doi: 10.1038/srep34866 (PMC5159873; doi:10.1038/srep34866)
Supplement: Supplementary Information [file srep34866-s2.pdf]

Proteomic analysis of scallop hepatopancreatic extract provides insights into marine polysaccharide digestion

Lyu Qianqian, Jiao Wenqian, Zhang Keke, Bao Zhenmin, Wang Shi, Liu Weizhi

Supplementary Table S2

List of protein accession numbers for phylogenetic analysis

ID_in_Phylogenetic_tree	Accession number or Gene ID
Cf_Fuca1	evm.model.scaffold15477.32
Cf_Fuca2	evm.model.scaffold63639.10.1
Cf_Fuca3	evm.model.scaffold34531.5
Cf_Fuca4	evm.model.scaffold41405.51
Cf_Fuca5	evm.model.scaffold63879.6
Cf_Fuca6	evm.model.scaffold63879.10
Cf_Fuca7	evm.model.scaffold36601.1
Cf_Fuca8	evm.model.scaffold63879.7
Cg_Fuca1	XP_011433349.1
Cg_Fuca2	XP_011435701.1
Cg_Fuca3	XP_011419679.1
Cg_Fuca4	XP_011419624.1
Cg_Fuca5	XP_011419621.1
Cg_Fuca6	XP_011419622.1
Cg_Fuca7	XP_011434194.1
Cg_Fuca8	XP_011417649.1
Cg_Fuca9	XP_011447776.1
Cg_Fuca10	XP_011416557.1
Cg_Fuca11	XP_011453881.1
Lg_Fuca1	XP_009048306.1
Lg_Fuca2	ESO90450.1
Lg_Fuca3	XP_009044265.1
Lg_Fuca4	XP_009049874.1
Lg_Fuca5	XP_009049999.1
Lg_Fuca6	XP_009050000.1
Lg_Fuca7	ESP01432.1
Ac_Fuca1	XP_005102742.1
Ac_Fuca2	XP_005102731.1
Ac_Fuca3	XP_005102732.1
Ac_Fuca4	XP_005102743.1
Ac_Fuca5	XP_005102744.1
Ac_Fuca6	XP_005102733.1
Ac_Fuca7	XP_012940107.1
Ac_Fuca8	XP_005102602.1
At_Fuca	NP_180377.2
Cf_ARSB1	evm.model.scaffold60269.41
Cf_ARSB2	evm.model.scaffold60269.45
Cf_ARSB3	evm.model.scaffold27081.2

Cf_ARSB4	evm.model.scaffold27081.4
Cf_ARSB5	evm.model.scaffold60269.44.1
Cf_ARSB6	evm.model.scaffold15949.4
Cf_ARSB7	evm.model.scaffold38493.31
Cg_ARSB1	XP_011446775.1
Cg_ARSB2	XP_011452356.1
Cg_ARSB3	EKC29950.1
Cg_ARSB4	XP_011457041.1
Cg_ARSB5	XP_011457045.1
Cg_ARSB6	XP_011416315.1
Cg_ARSB7	XP_011416314.1
Cg_ARSB8	XP_011422903.1
Lg_ARSB1	XP_009066363.1
Lg_ARSB2	XP_009066364.1
Lg_ARSB3	XP_009045786.1
Lg_ARSB4	XP_009058087.1
Lg_ARSB5	XP_009058088.1
Lg_ARSB6	XP_009049761.1
Lg_ARSB7	XP_009048365.1
Lg_ARSB8	XP_009049412.1
Lg_ARSB9	XP_009064931.1
Lg_ARSB10	XP_009059188.1
Lg_ARSB11	XP_009059189.1
Ac_ARSB1	XP_005090306.1
Ac_ARSB2	XP_012944880.1
Ac_ARSB3	XP_005090324.1
Ac_ARSB4	XP_005104849.1
Ac_ARSB5	XP_012941568.1
Ac_ARSB6	XP_012944884.1
Ac_ARSB7	XP_005090307.1
Ac_ARSB8	XP_005099114.1
Ac_ARSB9	XP_012939262.1
Ac_ARSB10	XP_005093145.1
Ac_ARSB11	XP_005099750.2
Ac_ARSB12	XP_012944441.1
Ac_ARSB13	XP_012937851.1
Ac_ARSB14	XP_005103162.1
Ac_ARSB15	XP_012940677.1
Ac_ARSB16	XP_012942173.1
Xt_ARSB	XP_002940244.2
Ng_ARSB	XP_005855624.1
